# Hsa_circ_0091581 promotes glioma progression by regulating RMI1 via sponging miR-1243-5p

**DOI:** 10.7150/jca.55558

**Published:** 2021-04-02

**Authors:** Jin Qian, Yingna Xu, Xing Xu, Zhenyu Tao, Yang Luo, Yichang Xu, Yong Zhang, Chunfa Qian

**Affiliations:** 1 Department of Neurosurgery, People's Hospital of Xuancheng City, Xuancheng, Anhui, China.; 2 Department of Neurosurgery, The Affiliated Brain Hospital of Nanjing Medical University, Nanjing, Jiangsu, China.

**Keywords:** circ_0091581, miR-1243-5p, RMI1, glioma.

## Abstract

Glioma is a pervasive malignancy and the main cause of cancer-related deaths worldwide. Circular RNA is an important subject of cancer research, and its role and function in glioma are poorly understood. This study demonstrated that hsa_circ_0091581 is upregulated in glioma tissues and cells. The results of the CCK-8, EdU, and transwell assays indicated that hsa_circ_0091581 promotes proliferation, migration, and invasion of glioma cells. The results of the luciferase reporter and RNA immunoprecipitation assays indicated that the mechanism of the effects of hsa_circ_0091581 on glioma cells involves sponging miR-1243-5p to regulate RMI1. The results of the rescue experiments indicated that hsa_circ_0091581 regulates proliferation, migration, and invasion of glioma cells by targeting RMI1 in a miR-1243-5p dependent manner. The results of the nude mice xenograft assays showed that knockdown of hsa_circ_0091581 inhibits glioma growth *in vivo*. Thus, our study determined the role of hsa_circ_0091581/miR-1243-5p/RMI1 in glioma and suggests that this axis may be a novel therapeutic target in glioma.

## Introduction

Glioma is a pervasive malignancy and the main cause of cancer-related deaths worldwide 1. Glioblastoma multiforme (GBM), WHO Ⅳ grade glioma, is characterized by the presence of poorly differentiated stromal cells around focal necrosis and capillary proliferation 2, 3. The latest statistics shows that GBM accounts for 12%~15% of all intracranial malignant tumors and 50%~60% of astrocytic tumors 4. Current systematic treatment strategies for glioma include surgery, chemotherapy, radiotherapy and tumor treating fields (TTF) and have introduced certain innovations; however, GBM grows rapidly, has a poor prognosis, and usually results in death within one year after diagnosis 5. Therefore, investigation of the molecular mechanisms of glioma occurrence and progression is very important.

Circular RNA (circRNA) is a specific type of RNA molecule and has been extensively investigated in the fields of RNA and disease research 6. CircRNA molecules differ from linear RNA (with 5' and 3' ends) and have a closed circular structure, which is not affected by RNA enzymes 7, 8. From the cell biology point of view, circRNAs are closely related to the biological functions of cells, such as proliferation, differentiation, and apoptosis 9, 10. Recent studies have shown that circRNAs play an important regulatory role in the growth and development of organisms and occurrence and development of diseases 11, 12. CircRNAs has attracted attention of the scientists in the field of cancer research. Numerous recent studies have suggested that circRNAs play an important role in tumor progression, and certain circRNAs can even be used as targets for early diagnosis, prognosis assessment and targeted therapy of tumors. For example, circ-ZKSCAN1 has been reported as a prognostic factor of bladder cancer recurrence 13; circ-UMAD1 in peripheral circulation may be a biomarker of lymph node metastasis in thyroid cancer 14; hsa_circ_0003829 may act as a diagnostic predictor in oral squamous cell carcinoma 15. In glioma, accumulating evidence indicates that circRNAs are closely associated with glioma progression 1, 16, 17; however, progress of the sequencing technology resulted in identification of additional circRNAs, and the relationships between circRNAs and glioma progression require further investigation.

In this study, we report that hsa_circ_0091581 is upregulated in glioma tissues and glioma cells. Function assays showed that hsa_circ_0091581 promotes proliferation, migration, and invasion of glioma cells. Using online public databases and RNA pull-down, RNA immunoprecipitation, and luciferase reporter assays, we determined the mechanism of the effect of hsa_circ_0091581 on glioma progression. Overall, our study is the first to report that hsa_circ_0091581 promotes glioma proliferation, migration, and invasion by targeting the miR-1243-5p/RMI1 axis that may be a potential target for glioma treatment.

## Methods

### Clinical samples

Glioma and corresponding adjacent nonneoplastic tissue samples (n=20) were obtained from patients diagnosed with glioma and admitted to the People's Hospital of Xuancheng City from June 2014 to September 2018. The samples were stored in liquid nitrogen immediately after resection. The pathological grade of the tumors was independently determined by two senior pathologists. Written informed consent was obtained from all patients. This study was approved by the Ethics Committee of People's Hospital of Xuancheng City.

### Cell culture and transfection

Glioma cell lines (U138, U87, U251, A172, and T98G) were obtained from the American Type Culture Collection (ATCC, MD, USA). Normal astrocytes were purchased from Procell (Wuhan, China). All cell lines were grown in Dulbecco's modified Eagle's medium (DMEM, Gibco, NY, USA) containing 10% fetal bovine serum (FBS, HyClone, UT, USA) and maintained at 5%CO_2_ and 37°C. The synthetic nucleotides and constructs used in this study were provided by GenePharma (Shanghai, China), and the transfection was performed using Lipofectamine 3000 (Invitrogen, CA, USA).

### Quantitative real-time PCR (qRT-PCR)

Total RNA was isolated from the cells or tissues by using TRIzol reagent (Invitrogen, CA, USA). Qualified RNA was reverse transcribed into complementary deoxyribonucleic acid (cDNA) using a PrimeScript RT kit (Invitrogen, CA, USA); qRT-PCR was performed using SYBR® Premix Ex Taq™ (TaKaRa, Tokyo, Japan) at 92°C for 10 min and 40 cycles at 92°C for 10 s and 60°C for 1 min. GAPDH was used as an internal control. The relative level of circ_0091581, miR-1243-5p, and RMI1 was calculated by the 2**^-ΔΔCt^** method. The primers used in this study were shown in **Table 1**.

### RNase R digestion assay

Isolated RNA (2 µg) was incubated with RNase R (3 U/µg) or digestion buffer at 37°C for 30 min. After the solution was purified, qPCR was performed to determine the RNA levels.

### Actinomycin D assay

Glioma cells were incubated with actinomycin D (3 µg/ml, Sigma, CA, USA) to block the transcription of mRNAs for 0 h, 8 h, 16 h, and 24 h. After cells were harvested, circular GPC3 (hsa_circ_0091581) and linear GPC3 RNAs were quantified by qRT-PCR to determine the half-life of RNA.

### Western bolt

Total protein was isolated by using RIPA lysis buffer (Keygene, Shanghai, China). After separation by SDS-PAGE, the proteins were transferred to PVDF membranes (Millipore, MA, USA). Then, the membrane was incubated with primary and secondary antibodies (Proteintech, IL, USA). Finally, the signals were detected by an Image Quant LAS 4000 system (GE, USA).

### Luciferase reporter assay

The synthetic nucleotide and constructs used in this assay were provided by GenePharma (Shanghai, China). The pmirGLO-circ_0091581-WT or pmirGLO-circ_0091581-MUT vectors were cotransfected with NC mimics or miR-1243-5p mimics into U87 and U251 cells. The pmirGLO-RMI1-WT or pmirGLO-RMI1-MUT vectors were cotransfected with NC mimics or miR-1243-5p mimics into U87 and U251 cells. Transfection was carried out by using Lipofectamine 3000 (Invitrogen, CA, USA). After 48 h, final luciferase activity was assessed using a luciferase reporter assay system (Promega, WI, USA).

### RNA immunoprecipitation (RIP)

RIP assay was performed using a Magna RIP RNA-binding protein immunoprecipitation kit (Millipore, MA, USA). The treated U87 and U251 cells were lysed in a lysis buffer containing protease and RNase inhibitors. Then, the cell lysates were incubated in a RIP buffer with magnetic beads conjugated with an anti-human Ago2 antibody (Millipore), and normal IgG (Millipore) were used as a negative control. Finally, the coprecipitated RNAs were eluted from the beads and assayed by qRT-PCR.

### Cell counting kit-8 (CCK-8) assay

Cells (2,000/well) were inoculated in a 96-well plate. At 12 h, 24 h, 48 h, and 72 h, the optical density at 450 nm at the indicated time was recorded using a CCK-8 kit (Beyotime, Shanghai, China), and viability curves were constructed.

### 5-Ethynyl-2-deoxyuridine (EdU) assay

Cells (20,000/well) were plated in a 96-well plate overnight. Then, the cells were incubated in 4% methanol for 30 min followed by permeabilization in 0.5% Triton X-100 (Keygene, Shanghai, China) for 10 min. Then, the cells were incubated with 1×ApollorR (RiboBio, Guangzhou, China) for 30 min. Cells were stained by 4',6-diamidino-2-phenylindole (DAPI) for another 30 min in the dark. Finally, EdU-positive cells were counted.

### Transwell assay

Cells (40,000/well) were suspended in DMEM without FBS and added to the upper section of a transwell chamber (8 µm; Millipore, MA, USA). DMEM containing FBS (10%) (600 µl) was added to the bottom of a 24-well plate with inserts. After culture for 48 h, the cells migrated or invaded to the bottom chamber were fixed and stained. In invasion assay, the chambers were precoated with Matrigel (200 mg/ml, BD, NJ, USA), and in migration assay, the precoating was not performed.

### Tumor xenograft assay

Eight female nude mice aged 5-6 weeks were purchased from Beijing Laboratory Animal Center (Beijing, China). U87 cells (1,000,000) transfected with sh-circ_0091581 or a negative control were injected subcutaneously into mice. Three weeks later, the tumors formed in mice were evaluated (equation: Volume=length×width^2^×0.5).

### Statistical analysis

The data are presented as the mean ± standard error and were analyzed by SPSS (Statistical Product and Service Solutions, Version 17.0, IL, USA). Figures were edited using GraphPad Prism (Version X; CA, USA). Two-paired independent t-test was performed to assess the differences between the groups. Differences were considered significant at *P*< 0.05.

## Results

### The expression and characteristics of hsa_circ_0091581 in glioma

**Figure 1:**
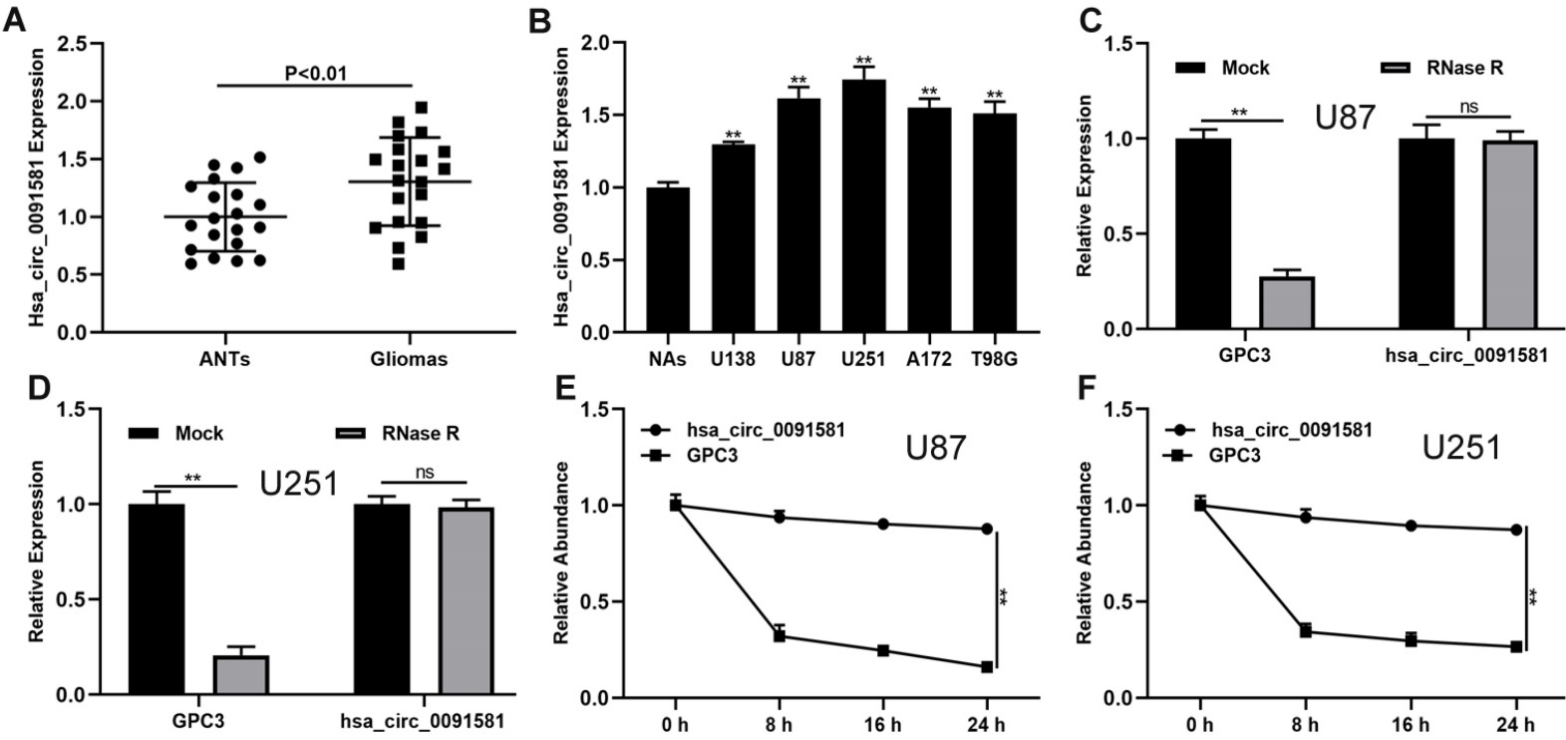
** Hsa-circ_0091581 is upregulated in glioma tissues and cells. (A)** The expression of hsa_circ_0091581 in glioma and adjacent nonneoplastic tissue (n=20). **(B)** The expression of hsa_circ_0091581 in glioma cells. **(C-D)** The RNase R assay confirmed the circular structure of hsa_circ_0091581 in U87 and LN229 cells. **(E-F)** Circular transcript of GPC3 (hsa_circ_0091581) was more stable than linear transcript according to the results of the actinomycin D treatment assays in U87 and LN229 cells.

The expression of hsa_circ_0091581 in glioma and the corresponding adjacent nonneoplastic tissue (ANTs) was assayed by qRT-PCR. Compared with ANTs, hsa_circ_0091581 was significantly upregulated in glioma tissues **(Fig. 1A)**. Moreover, compared with normal astrocytes (NAs), hsa_circ_0091581 was considerably upregulated in glioma cells **(Fig. 1B)**. To validate the circular characteristics of hsa_circ_0091581, U87 and U251 cells were treated with RNase R. The results showed that RNase R did not digest hsa_circ_0091581** (Fig. 1C, D)**. Furthermore, the results of the actinomycin D assay indicated that the circular transcript (hsa_circ_0091581) is more stable than the linear transcript (GPC3) in U87 and U251 cells **(Fig. 1E, F)**. There results suggest that hsa_circ_0091581 may have important functions in glioma.

### Hsa_ circ_0091581 promotes the proliferation, migration, and invasion of glioma cells *in vitro*

**Figure 2:**
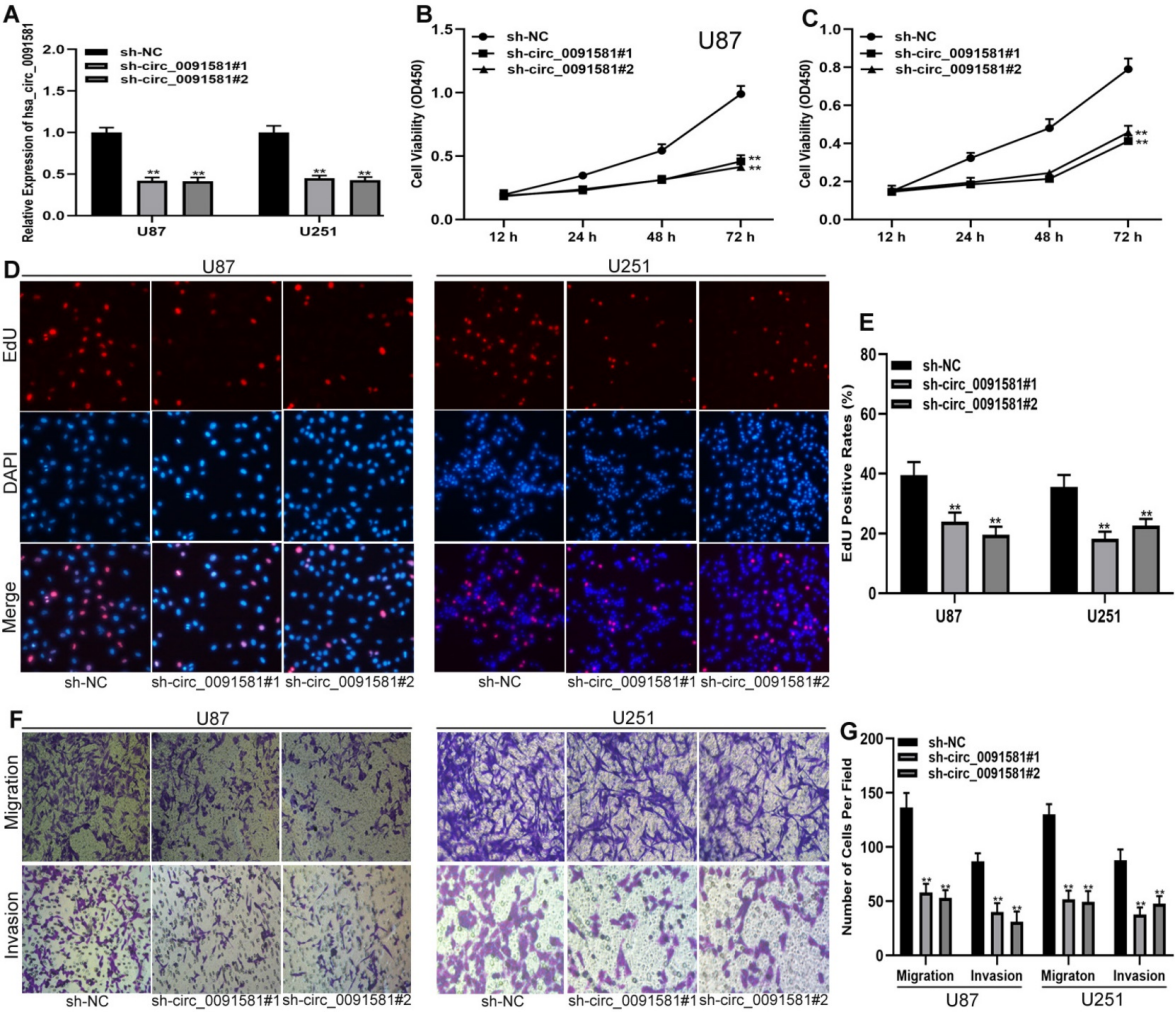
** Downregulation of hsa-circ_0091581 inhibits the proliferation, migration, and invasion of glioma cells. (A)** qRT-PCR assay of the expression of hsa_circ_0091581 and GPC3 in U87 and U251 cells transfected with sh-NC or sh-circ_0091581. **(B-C)** Cell proliferation was assessed by CCK-8 assay after hsa_circ_0091581 downregulation in U87 and U251 cells. **(D-E)** Cell proliferation was assessed by EdU assay after hsa_circ_0091581 downregulation in U87 and U251 cells. **(F-G)** Cell migration and invasion were evaluated by transwell assay after hsa_circ_0091581 downregulation in U87 and U251 cells.

To test the functions of hsa_circ_0091581 in glioma cells, short hairpin RNAs (shRNAs) and corresponding negative control were used to construct three cell models with various expression levels of hsa_circ_0091581 **(Fig. 2A)**. CCK-8 assay showed that hsa_circ_0091581 knockdown inhibits the proliferation of U87 and U251 cells **(Fig. 2B, C)**. The results of the EdU assay are similar to that of the CCK-8 assay** (Fig. 2D, E)**. The results of the transwell assay indicated that hsa_circ_0091581 downregulation suppresses the migration and invasion of U87 and U251 cells **(Fig. 2F-G)**.

### Hsa_ circ_0091581 functions as a sponge of miR-1243-5p

**Figure 3:**
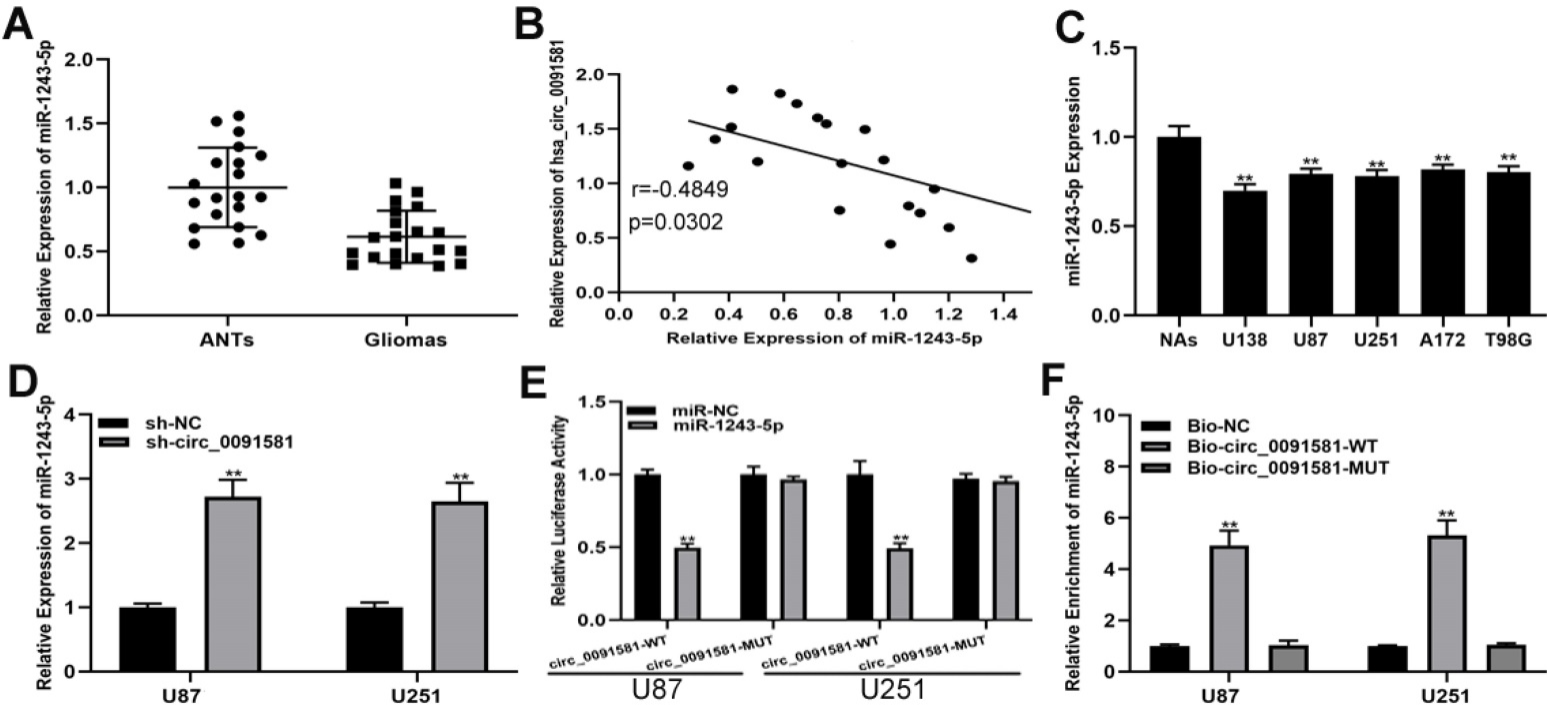
** Hsa_circ_0091581 sponges miR-1243-5p in U87 and U251 cells. (A)** The expression of miR-1243-5p in glioma and adjacent nonneoplastic tissue (n=20). **(B)** Pearson correlation analysis indicated a reverse correlation between hsa_circ_0091581 and miR-1243-5p. **(C)** The expression of miR-1243-5p in glioma cells. **(D)** qRT-PCR assay of the expression of miR-1243-5p in U87 and U251 cells transfected with sh-NC or sh-circ_0091581. **(E)** The relative luciferase activity was assayed in U87 and U251 cells cotransfected with miR-1243-5p mimics or miR-NC and hsa_circ_0091581 wild type or mutant luciferase reporter vectors. **(F)** Biotinylated RIP assay of the cells transfected with Bio-circ_0091581, Bio-circ_0091581-MT, or a control vector.

A number of studies have shown that circRNAs can regulate the progress of glioma by adsorbing miRNAs, which called competing endogenous RNAs (ceRNA) mechanism 18, 19. An online database (circInteractome, https://circinteractome.nia.nih.gov/) was searched to selected 28 miRNAs as the sponge targets of hsa_circ_0091581. miRNAs with the top ten scores were selected for further screening, and miR-1243-5p was of interest. Initially, we detected the expression of miR-1243-5p in the clinical samples and found that miR-1243-5p was downregulated in glioma versus ANTs **(Fig. 3A)**. Pearson correlation analysis indicated an inverse correlation between hsa_circ_0091581 and miR-1243-5p **(Fig. 3B)**. Predictably, comparison with NAs indicated that miR-1243-5p is downregulated in glioma cells **(Fig. 3C)**. Additionally, qRT-PCR showed that knockdown of hsa_circ_0091581 can upregulate miR-1243-5p in U87 and U251 cells **(Fig. 3D)**. Moreover, the results of the luciferase reporter assays indicated that miR-1243-5p can decrease the luciferase activity in the case of hsa_circ_0091581-WT and has no effect on the expression of hsa_circ_0091581-MUT **(Fig. 3E)**. Finally, the results of the RIP assay indicated that miR-1243-5p is enriched in the Bio-hsa_circ_0091581 group **(Fig. 3F)**. These results demonstrated that hsa_circ_0091581 can function as a sponge of miR-1243-5p.

### RMI1 is the functional target of miR-1243-5p

**Figure 4:**
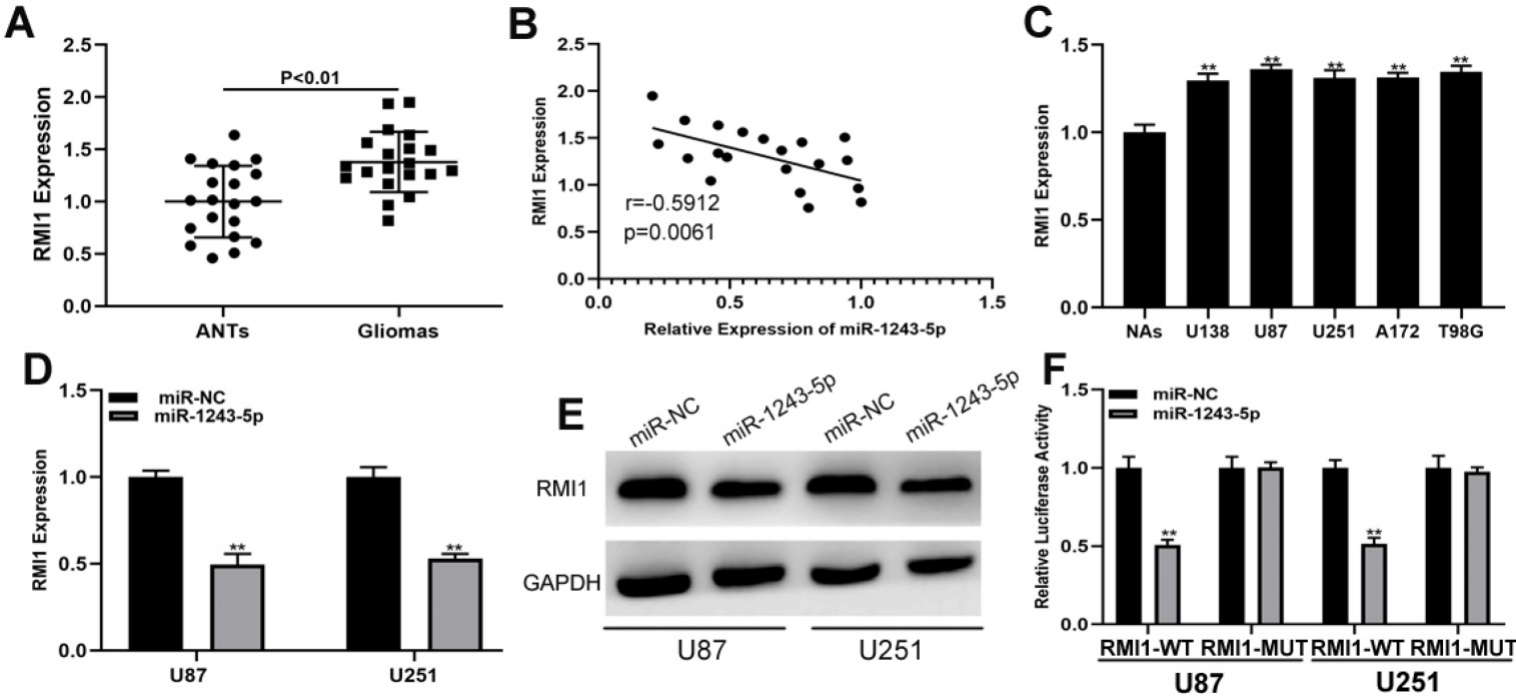
** RMI1 is the functional target of miR-1243-5p in U87 and U251 cells. (A)** The expression of RMI1 in glioma and adjacent nonneoplastic tissue (n=20). **(B)** Pearson correlation analysis indicated a reverse correlation between RMI1 and miR-1243-5p. **(C)** The expression of RMI1 in glioma cells. **(D-E)** qRT-PCR and western blot assays of the expression of RMI1 in U87 and U251 cells transfected with miR-NC or miR-1243-5p mimics. **(F)** The relative luciferase activity was assayed in U87 and U251 cells cotransfected with miR-1243-5p mimics or miR-NC and RMI1 wild type or mutant luciferase reporter vectors.

The targets of miR-1243-5p were identified by screening an online database (targetScan, http://www.targetscan.org/vert_72/), and RMI1 was selected for further investigations because of its high score. The expression of RMI1 in clinical samples indicated that RMI1 was upregulated in glioma tissues compared with the level in ANTs **(Fig. 4A)**. The result of Pearson correlation analysis showed that RMI1 is negatively associated with miR-1243-5p **(Fig. 4B)**. Additionally, the expression of RMI1 was higher in glioma cells than that in NAs **(Fig. 4C)**. Furthermore, the results of the qRT-PCR and western blot assays indicated that miR-1243-5p can repress RMI1 expression in U87 and U251 cells **(Fig. 4D, E)**. Finally, the results of the luciferase reporter assay indicated that miR-1243-5p can decrease the luciferase activity in the case of RMI1-WT and has no effect on the expression of RMI1-MUT **(Fig. 4F)**. Overall, these results indicated that RMI1 is the function target of miR-1243-5p.

### Hsa_ circ_0091581 regulates the proliferation, migration, and invasion of glioma cells in a miR-1243-5p-dependent manner

**Figure 5:**
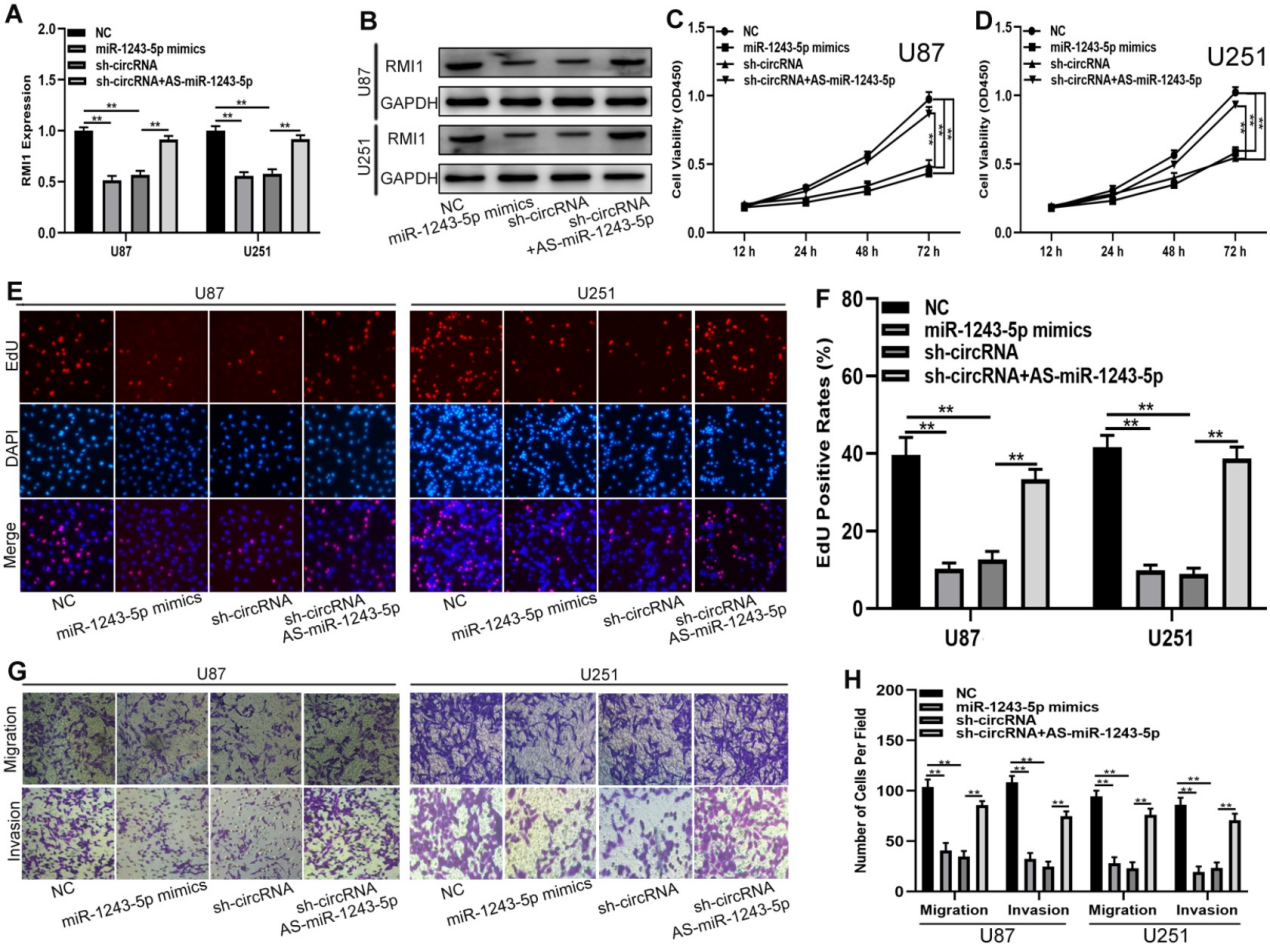
** Hsa_circ_0091581 promotes the proliferation, migration, and invasion of glioma cells by targeting RMI1 in a miR-1243-5p dependent manner. (A-B)** qRT-PCR and western blot assay of the expression of RMI1 in U87 and U251 cells transfected with sh-NC+miR-NC, sh-NC+miR-1243-5p mimics, miR-NC+sh-circ_0091581, or sh-circ_0091581+miR-1243-5p inhibitors. **(C-D)** CCK-8 assay results indicate that miR-1243-5p mimics and sh-circ_0091581 inhibit the proliferation of U87 and U251 cells, and the inhibitory effect of sh-circ_0091581 is restored by miR-1243-5p inhibitors. **(E-F)** EdU assay results indicate that miR-1243-5p mimics and sh-circ_0091581 inhibit the proliferation of U87 and U251 cells, and the inhibitory effect of sh-circ_0091581 is restored by miR-1243-5p inhibitors. **(G-H)** Transwell assay results indicate that miR-1243-5p mimics and sh-circ_0091581 inhibit the migration and invasion of U87 and U251 cells, and the inhibitory effect of sh-circ_0091581 is restored by miR-1243-5p inhibitors.

To determine whether the function of hsa_circ_0091581 in glioma cells is dependent on the miR-1243-5p/RMI1axis, shRNAs, miR-1243-5p mimics, and miR-1243-5p inhibitors were used to construct four cell models. The expression of RMI1 in the four cell models was tested by qRT-PCR and western blot **(Fig. 5A, B)**. The results of the CCK-8 and EdU assays showed that miR-1243-5p overexpression and hsa_circ_0091581 downregulation can suppress the proliferation of U87 and U251 cells, and downregulation of miR-1243-5p can restore the inhibitory effect of hsa_circ_0091581 downregulation on the proliferation **(Fig. 5C-F)**. Moreover, the results of the transwell assay indicated that upregulation of miR-1243-5p and knockdown of hsa_circ_0091581 can inhibit the migration and invasion of U87 and U251 cells, and the inhibitory effect of hsa_circ_0091581 can be rescued by miR-1243-5p inhibitors **(Fig. 5G, H)**. These findings suggested that hsa_circ_0091581 regulates the proliferation, migration, and invasion of glioma cells by targeting RMI1 in a miR-1243-5p dependent manner.

### Knockdown of hsa_circ_0091581 inhibits glioma growth in a nude mice xenograft model

**Figure 6:**
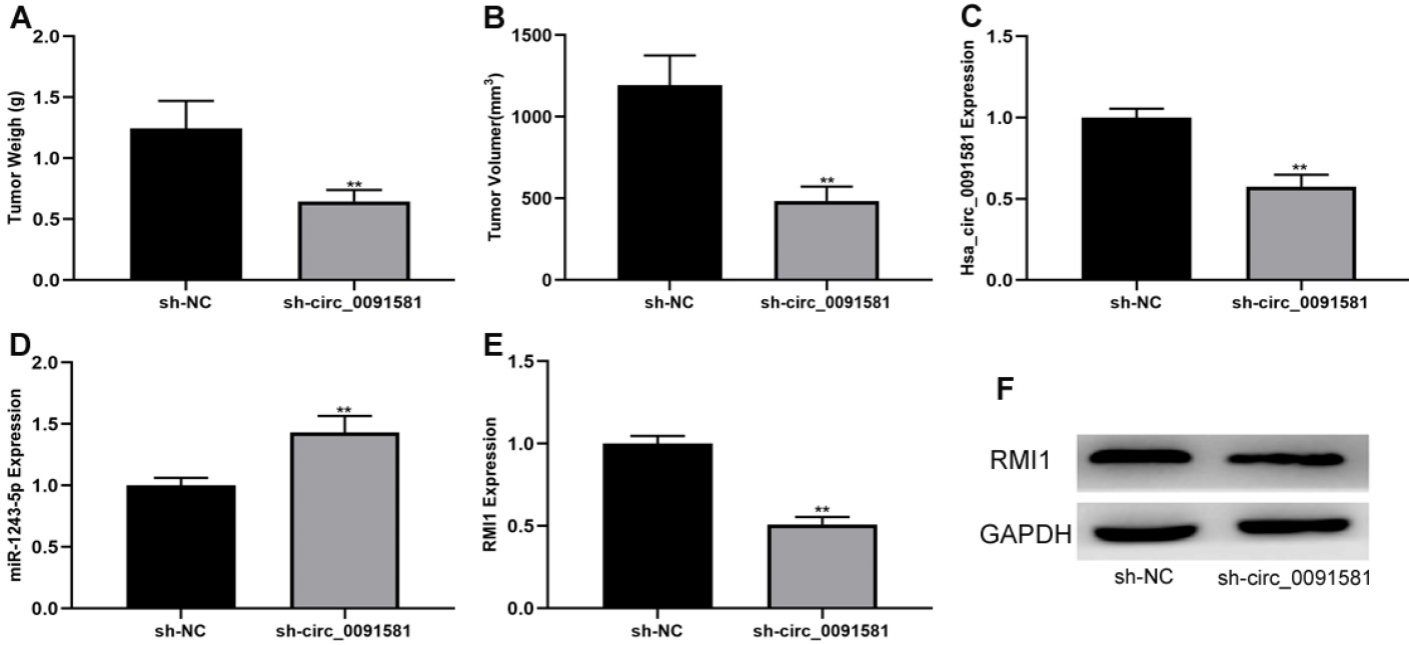
** Hsa_circ_0091581 promotes glioma growth *in vivo.* (A-B)** Downregulation of hsa_circ_0091581 decreases tumor growth* in vivo*. **(C)** The expression of hsa_circ_0091581 in the tumors formed in the two groups of mice. **(D)** Downregulation of hsa_circ_0091581 increases miR-1243-5p expression in the tumor. **(E-F)** Downregulation of hsa_circ_0091581 reduces RMI1 expression in the tumor.

To determine whether hsa_circ_0091581 can suppress glioma growth *in vivo*, a nude mice xenograft model was generated. U87 cells (1,000,000) transfected with sh-circ_0091581 or negative control were injected into the right shoulder of mice. After 21 days, the tumors formed in mice were evaluated. The results showed that tumors formation in the sh-circ_0091581 group had lower weight and volume compared with those in the negative control group **(Fig. 6A, B)**. Moreover, the results of qRT-PCR and/or western blot indicated that hsa_circ_0091581 and RMI1 were downregulated and miR-1243-5p was upregulated in sh-circ_0091581 group compared with those in the negative control group** (Fig. 6C-F)**. Thus, knockdown of hsa_circ_0091581 inhibits glioma growth *in vivo*, and this effect is associated with miR-1243-5p and RMI1.

## Discussion

Glioma has always been an important subject of research and a challenge for neurosurgery 20, 21. At present, the treatment of glioma mainly includes surgical resection followed by adjuvant radiotherapy and chemotherapy 22. Recently, a variety of new treatment methods of glioma have emerged and are used in clinic to improve the prognosis of patients to an extent; however, the median survival time of glioma patients has not improved significantly^23^. Thus, investigation of the molecular mechanisms of glioma pathogenesis and progression is important. Rapid development of next generation sequencing (NGS) technology results in detection of an increasing number of noncoding RNAs (ncRNAs) and especially circRNAs 24. circRNAs play an important role in the development of human diseases. For example, hsa_circ_0026416 acts as an oncogene in colorectal cancer 25; circCFL1 can promote the progression of diffuse large B-cell lymphoma 26; and hsa_circ_0001068 can be a novel biomarker of ovarian cancer 27. Hsa_circ_0091581 is a novel circRNA transcribed from the glypican-3 gene (GPC3). hsa_circ_0091581 plays an oncogenic role in hepatocellular carcinoma^28^, ^29^; however, its role in glioma was not investigated previously. In present study, we show that hsa_circ_0091581 is upregulated in glioma tissues and cell lines and can promote the proliferation, migration, and invasion of glioma cells *in vitro*.

To determine the mechanism of action of hsa_circ_0091581 on glioma proliferation, migration, and invasion, we used the results described in the literature that suggested that circRNAs can play a regulatory role by adsorbing miRNAs 30-32. Hsa_circ_0001785 sponges miR-942 to regulate breast cancer progression 33; hsa_circ_0005556 sponges miR-4270 to facilitate gastric cancer progression 34; and hsa_circ_0004913 act as a sponge for miR-184 and is thus involved in the progress of hepatocellular carcinoma 35. Ji and Wei et al. reported that hsa_circ_0091581 is involved in hepatocellular carcinoma progression by sponging miR-591 and miR-526b 28, 29. In the present study, hsa_circ_0091581 was able to sponge miR-1243-5p involved in the progression of pancreatic cancer as a target of hsa_circ_0002570 36. Then, a search of an online database and luciferase reporter assays were used to select RecQ-mediated genome instability protein 1 (RMI1) as the target of miR-1243-5p. RMI1 plays an important role in DNA repair and is closely associated with many human diseases, including tumors, obesity, and Bloom syndrome 37-39. However, the role of RMI1 in glioma has not been reported previously. Our data indicate that RMI1 is upregulated in glioma tissues and cell lines, and qRT-PCR, western blot, and functional assays demonstrated that RMI1 is a functional target of miR-1243-5p. Additionally, rescue experiments suggested that hsa_circ_0091581 regulates the expression and function of RMI1 in a miR-1243-5p dependent manner. Finally, *in vivo* assays suggested that hsa_circ_0091581 can inhibit glioma growth, and this effect was mediated by miR-1243-5p and RMI1.

In summary, hsa_circ_0091581 can promote glioma proliferation, migration, and invasion via the hsa_circ_0091581/miR-1243-5p/RMI1 axis and may be a novel therapeutic target in glioma.

## Figures and Tables

**Table 1:** The primers used in this study.

Gene	Forward	Reverse
Circ_0091581	GGAGAACGTACTGCTTGGTC	TGGAGTCAGGCTTGGGTAGT
miR-1243-5p	GTCAACTGGATCAATTATAGG	GTGCAGGGTCCGAGGT
RMI1	GTGCGATCCTCAAGAGCGTA	CAGACATCCATCAGCCGGAC
GAPDH	TCGACAGTCAGCCGCATCTTCTTT	ACCAAATCCGTTGACTCCGACCTT
U6	CTCGCTTCGGCAGCACA	AACGCTTCACGAATTTGCGT
